# Immersive medium for early clinical exposure – knowledge acquisition, spatial orientation and the unexpected role of annotation in 360° VR photos

**DOI:** 10.3205/zma001590

**Published:** 2023-02-15

**Authors:** Robert Speidel, Achim Schneider, Steffen Walter, Claudia Grab-Kroll, Wolfgang Oechsner

**Affiliations:** 1 Ulm University, Medical Faculty, Division of Learning and Teaching, Competence Center eEducation in Medicine, Ulm, Germany; 2 Ulm University, Medical Faculty, Division of Learning and Teaching, Ulm, Germany; 3 University Hospital Ulm, Department of Medical Psychology, Ulm, Germany; 4 University Hospital Ulm, Clinic for Anesthesiology and Intensive-Care Medicine, Ulm, Germany

**Keywords:** 360° photos, 360° VR photos, virtual reality, learning, orientation, annotation

## Abstract

**Aim:**

360° VR photos could be a low-threshold possibility to increase early clinical exposure. Apart from granting insights into local routines and premises, the medium should facilitate knowledge acquisition and spatial orientation depending on its design. This assumption, however, is not yet substantiated empirically. Thus, three hypotheses were tested in consideration of Mayer’s modality principle: 1) Providing 360° VR photos as visual reference improves retention and comprehension of information. 2) The annotation of text boxes in 360° VR photos compromises spatial orientation and presence. 3) Annotated audio commentary is superior to annotated text boxes in terms of cognitive load and knowledge acquisition.

**Methods:**

Using head-mounted displays, students of human (N=53) and dental medicine (N=8) completed one of three virtual tours through a surgical unit, which were created with 360° VR photos. In the first two variants, information about the facilities, medical devices and clinical procedures was annotated either as text boxes or audio commentary comprising 67 words on average (SD=6.67). In the third variant, the same information was given separately on a printed handout before the virtual tour. Taking user experience and individual learner characteristics into account, differences between conditions were measured regarding retention, comprehension, spatial orientation, cognitive load, and presence.

**Results:**

Concerning retention and comprehension of information, annotated text boxes outperformed annotated audio commentary and the handout condition. Although annotated audio commentary exhibited the lowest knowledge test scores, students preferred listening over reading. Students with an interest in VR and 360° media reported higher levels of enjoyment and presence. Regarding spatial orientation and presence, no significant group differences were found.

**Conclusions:**

360° VR photos can convey information and a sense of spatial orientation effectively in the same learning scenario. For students, their use is both enjoyable and instructive. Unexpectedly, the ideal mode of annotation is not dictated by Mayer’s modality principle. For information like in this study, annotated text boxes are better for knowledge acquisition than the subjectively preferred audio commentary. This finding is probably contingent on the length and the quality of the annotated text. To identify boundary conditions and to validate the findings, more research is required on the design and educational use of 360° VR photos.

## Zusammenfassung

**Zielsetzung:**

360°-VR-Fotos könnten eine niedrigschwellige Möglichkeit sein, die klinische Exposition in den frühen Phasen der medizinischen Ausbildung zu steigern. Das Medium soll nicht nur Einblicke in lokale Räumlichkeiten und Abläufe bieten, sondern auch abhängig von der Annotationsform den Wissenserwerb und die räumliche Orientierung fördern. Empirisch belegt ist diese Annahme allerdings noch nicht. Daher wurden unter Berücksichtigung des Modalitätsprinzips nach Mayer drei Hypothesen getestet: 1) Der Einsatz von 360°-VR-Fotos als visuelle Referenz verbessert das Erinnern und Verstehen von Informationen. 2) Die Annotation von Textfeldern in 360°-VR-Fotos beeinträchtigt die räumliche Orientierung und Präsenz. 3) Annotierte Audiokommentare sind annotierten Textfeldern in Bezug auf die kognitive Belastung und den Wissenserwerb überlegen.

**Methodik:**

Mittels VR-Brillen absolvierten Studierende der Human- (N=53) und Zahnmedizin (N=8) einen von drei virtuellen Rundgängen durch einen OP-Bereich, die mit 360°-VR-Fotos erstellt wurden. In den ersten beiden Varianten wurden Informationen zu den Räumlichkeiten, medizinischen Geräten und klinischen Verfahren entweder in Form von Textfeldern oder Audiokommentaren mit durchschnittlich 67 Wörtern (SD=6,67) annotiert. In der dritten Variante wurden dieselben Informationen separat vor dem virtuellen Rundgang auf einem gedruckten Handout ausgeteilt. Unter Berücksichtigung individueller Lernereigenschaften und der Benutzererfahrung wurden Unterschiede zwischen den Bedingungen hinsichtlich des Erinnerns, des Verstehens, der räumlichen Orientierung, der kognitiven Belastung und der Präsenz gemessen.

**Ergebnisse:**

Hinsichtlich des Erinnerns und Verstehens von Informationen schnitten die annotierten Textfelder besser ab als die annotierten Audiokommentare und die Handout-Bedingung. Obwohl die annotierten Audiokommentare die niedrigsten Werte im Wissenstest aufwiesen, zogen die Studierenden das Hören dem Lesen vor. Studierende mit Interesse an VR und 360°- Medien berichteten über ein höheres Maß an Vergnügen und Präsenz. Hinsichtlich der räumlichen Orientierung und Präsenz wurden keine signifikanten Gruppenunterschiede festgestellt.

**Schlussfolgerung:**

360°-VR-Fotos können Informationen und ein Gefühl der räumlichen Orientierung effektiv in einem Lernszenario vermitteln. Für die Studierenden ist ihre Verwendung unterhaltsam und lehrreich. Die ideale Art der Annotation wird wider Erwarten nicht durch Mayers Modalitätsprinzip vorgeschrieben. Für Informationen wie die in dieser Studie eignen sich annotierte Textfelder besser zum Wissenserwerb als die subjektiv bevorzugten Audiokommentare. Dieses Ergebnis ist wahrscheinlich abhängig von der Länge und Qualität des annotierten Textes. Um Randbedingungen zu identifizieren und die Ergebnisse zu validieren, sind weitere Untersuchungen zur Gestaltung und didaktischen Nutzung von 360°-VR-Fotos erforderlich.

## 1. Introduction

### 1.1. Clinical exposure through 360° VR photos

The call for early clinical exposure slowly disbands the rigid sequence from preclinical to clinical education [1]. Yet, providing clinical exposure throughout the curriculum is a challenge for medical faculties, especially in regard of the rising number of medical students [2], [3]. Virtual reality (VR) could be a remedy even if its didactic potential is not yet fully acknowledged among lecturers and students alike [4]. Steve Bryson defines the technology as “the use of three-dimensional displays and interaction devices to explore real-time computer-generated environments” [5]. His definition applies to a range of different hardware but nowadays head-mounted displays (HMDs) are the most common association. When running VR software, HMDs isolate the user from reality and simultaneously render an environment depending on the user’s movements and actions, thereby creating an immersive illusion of an alternate, virtual reality. If the illusion succeeds, the user experiences a feeling of “presence”, i.e. the impression of actually being in the displayed environment [6]. In this mental state, the individual experience and behavior in VR resembles that in a real situation [7]. 

In medical education, VR could allow students to assume the role of a physician and treat virtual patients from their first semester onwards. However, the generic clinical wards and treatments of freely available VR software do not depict local hospitals with their unique premises, clinical routines, and medical specializations. If medical educators want to grant an immersive, early glimpse into regional practices, they require their own custom VR environments. At present, the development of VR software requires extensive financial and human resources in form of programmers, 3D-artists, and subject-matter experts. A budget alternative to creating custom, computer-generated VR environments are 360° media, which can be produced by medical educators themselves with little technical knowledge required.

**Figure 1:**
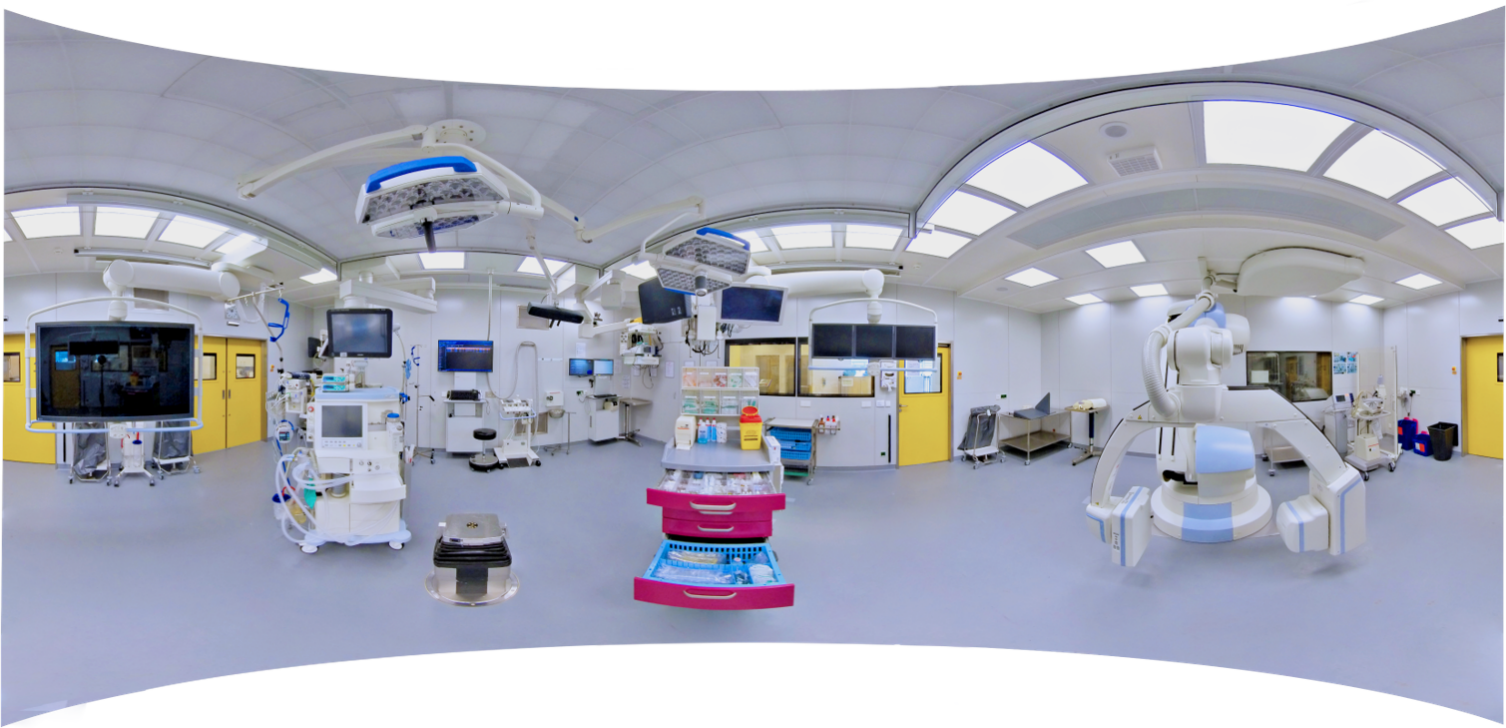
360° photo of an operating room. The 360° photo provides an omnidirectional view on an operating room of the University Clinic in Ulm. If displayed spherically on an HMD, users can explore the then called 360° VR photo without distortion through movements of the head (see figure 2).

**Figure 2:**
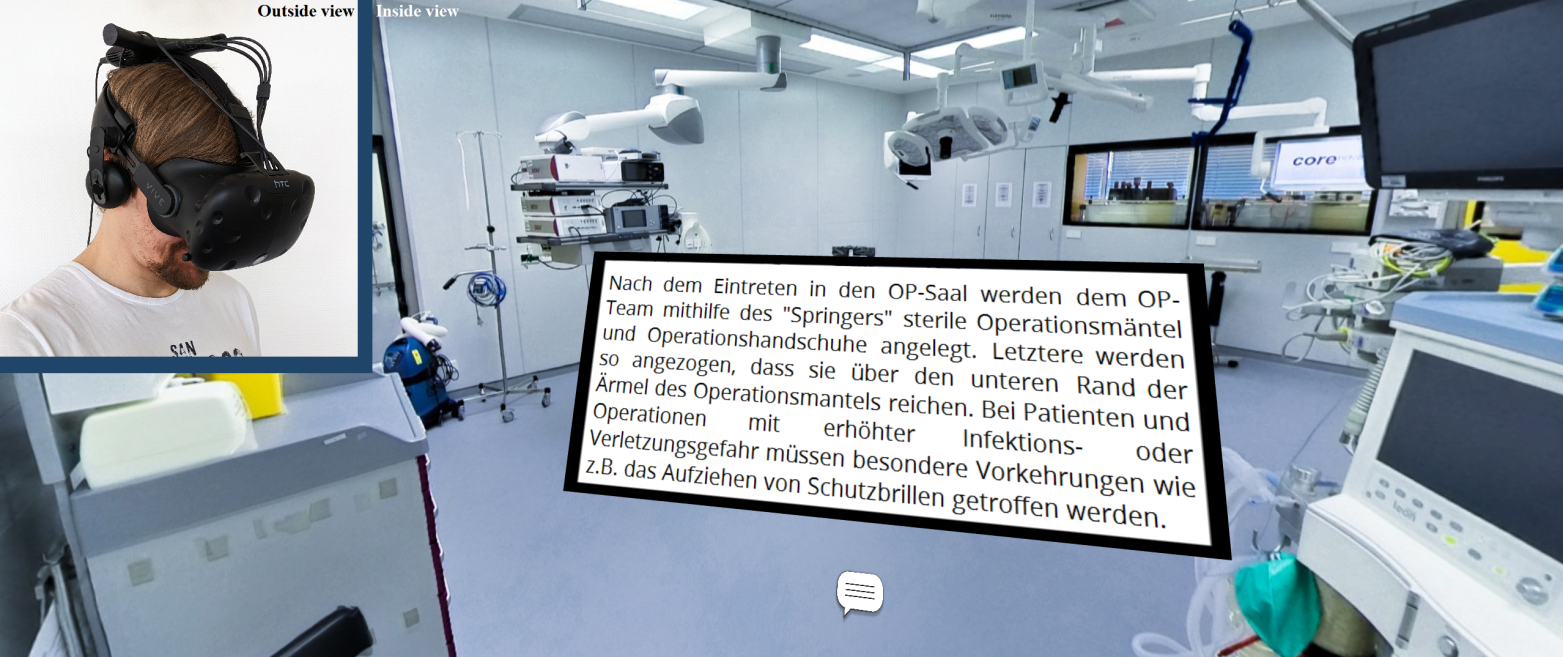
Study participant reading text (in German) in the virtual tour. The photo in the top left corner shows a study participant wearing a HTC Vive HMD. The adjacent image is a cropped screenshot of the virtual tour with an annotated text box above the corresponding hotspot.

The term “360° media” refers to both 360° videos and 360° photos which provide an omnidirectional view of a recorded environment. While 360° videos may depict action, 360° photos are static (see figure 1 (Fig. 1)). Both types of 360° media are produced with 360° cameras, which currently range between a hundred to a few thousand euros depending on the desired visual quality. In contrast to real-time computer-generated VR, 360° media restrict spatial movement to the position of the 360° camera and reduce interaction to so-called “hotspots”. Hotspots are visual control elements added in post-production which allow users to teleport between viewpoints and to display additional information in form of textual (text boxes or audio commentary) or graphical (e.g. close-ups or 3D models) annotations. 360° media which are linked and annotated through hotspots are referred to as “virtual tours” and can be viewed on all common display types (desktop, laptop, tablet, smartphone, or HMD). If viewed on an HMD (see figure 2 (Fig. 2)), virtual tours induce the VR characteristic feeling of presence [8], even though they do not fully comply with Bryson’s VR definition. The combination of 360° media and HMDs is therefore considered a variation of VR [9] and referred to as “360° VR media”. 360° videos and 360° photos which are displayed on HMDs are called “360° VR videos” and “360° VR photos”, accordingly. This terminology corresponds with previous research in the field [10], [11].

360° VR media allow medical students to virtually attend surgeries and explore locations with restricted access such as surgical and intensive care units in an immersive manner and independent of timetables and teaching personnel. While both 360° VR videos and 360° VR photos could potentially facilitate early clinical exposure, this study focuses specifically on 360° VR photos. However, the following chapter references findings on 360° VR videos too, because educational research on 360° VR photos in particular is scarce.

#### 1.2. Learning objectives in 360° VR media

Previous research ascribes a high didactic value to real-time computer-generated VR [12], [13], [14]. However, the referenced meta-analyses do not apply to 360° VR media automatically because camera recordings prohibit free spatial movement and direct object interaction. While these constraints do not reduce learning effectiveness necessarily [15], they prevent users to perform manual tasks in the virtual environment, limiting the application of 360° VR media for educational purposes. In fact, it is still unclear which learning objectives may be facilitated by 360° VR media as relevant research is still rare. In this study, we consider the general learning objectives of knowledge acquisition and spatial orientation.

According to Anderson and Krathwohl’ s revision of Bloom et al.’s taxonomy [16], [17], knowledge acquisition can be differentiated along two dimensions. The first dimension refers to four categories of attainable knowledge: Factual, conceptual, procedural, and metacognitive (examples in chapter 2.2). The second dimension refers to six levels of cognitive processing: Remember, understand, apply, analyze, evaluate, and create. Together, the two dimensions describe specific goals in knowledge acquisition (e.g. *remembering* the *factual* ratio of a child’s bodyweight to the amount of energy required in defibrillation). So far, educational research on 360° VR media mostly lacks this differentiation. Concerning 360° VR videos, studies have shown that the medium is only similarly effective as conventional videos and traditional lessons for acquiring knowledge of uncategorized nature [18], [19], [20], albeit being associated with increased levels of interest, engagement and enjoyment among students [11], [18], [19]. For 360° VR photos, educational studies are even rarer and currently limited to the field of construction management and safety education. In a study by Kim et al. [21], participants learned procedural information about construction activities with 360° VR photos. Compared to learning with 360° photos on smartphones, the 360° VR photos subjectively improved knowledge acquisition and were recommended to supplement or even replace actual field trips by most in the sample. Another argument for the didactic application of 360° VR photos is their immersive quality. According to a study by Krokos et al. spatially represented information is recalled more readily if it is acquired while wearing an HMD instead of sitting in front of a conventional display [22].

The second learning objective investigated in this study is spatial orientation, which refers to the ability to identify the relative location and direction of oneself and objects in an environment. Spatial orientation is required to contemplate action and to navigate to a desired destination [23]. Google Street View, which is available on conventional devices such as tablets and smartphones, is a prominent example for regular 360° photos being used in everyday life for spatial orientation. The use of HMDs has not been found to improve spatial orientation performance [24], [25], but it allows to explore distant or inaccessible locations in an immersive and natural manner through movements of the head. Moreover, research with rodents showed that the cognitive processing associated with spatial orientation is similar between real-time computer-generated VR and real environments [26]. Again, these findings do not apply to 360° VR photos automatically because they restrict locomotion to teleporting between viewpoints. Compared to actual walking, teleportation might compromise spatial orientation [25], [27], [28], [29]. This concern, however, does not discount the potential of 360° VR photos for exploring clinical environments when real site visits are not an option. 

In short, theory and preceding research suggest that 360° VR photos should be beneficial for both spatial orientation and knowledge acquisition. This assumption, however, is not yet substantiated empirically. The present study therefore investigates whether information can be learned effectively in annotated 360° VR photos, without compromising the learner’s spatial orientation and presence. To check for boundary conditions, the findings are analyzed and interpreted in consideration of individual learner characteristics (spatial orientation ability, affinity for information technology, prior experience with VR, interest in VR and 360° media) and the user experience (cognitive load, presence, simulation sickness, enjoyment, preferred mode of annotation, subjective didactic value of 360° VR photos). These factors provide additional information about the educational application of 360° VR photos. For example, interest and prior experience in VR are associated with a more favourable appraisal of the technology and reduced simulation sickness [30], [31], [32], [33]. Simulation sickness can compromise the learning experience through symptoms of fatigue, disorientation and nausea [34].

#### 1.3. Modality principle

To determine the most efficient and least disruptive mode of annotation, the validity of the modality principle was tested in the context of 360° VR photos [35], additionally. The modality principle derives from Mayer’s cognitive theory of multimedia learning and states that learning with graphics such as videos and photos is more prolific if the graphics are accompanied with audio commentary instead of written text. In theory, processing graphics and text simultaneously may overload the visual channel, whereas listening to spoken commentary utilizes the auditory channel too, thereby reducing the overall cognitive load [36]. This principle has been verified for computer-based learning [37], but previous research only considered graphics in a rectangular format on non-immersive displays (e.g. physiological functioning of the kidney illustrated on a desktop screen [38]). The principle is expected to apply to 360° VR photos too, because the omnidirectional view through an HMD should constitute a high strain for the visual channel, resulting in compromised reading comprehension and retention. As text boxes partially block the field of view, audio commentary is also expected to be preferable for spatial orientation and the feeling of presence.

#### 1.4. Hypotheses

Bearing these considerations in mind, three hypotheses were tested.

1. Providing 360° VR photos as visual reference improves retention and comprehension of information.
2. The annotation of text boxes in 360° VR photos compromises spatial orientation and presence.
3. Annotated audio commentary is superior to annotated text boxes in terms of cognitive load and knowledge acquisition.

## 2. Methods

### 2.1. Sample

To test the hypotheses, medical students were asked via e-mail and blog posts to voluntarily partake in a virtual tour through the surgical unit of the University Clinic in Ulm. After giving informed consent in writing, 61 students, who were enrolled in either human (*N*=53) or dental medicine (*N*=8), eventually participated in the study. As an incentive, each student received a 30€ voucher for their participation. None of the students had received medical education outside of their studies and had visited an operating theatre before. The participants were predominantly female (71%), mostly in their early twenties (*M*=21.79, *SD*=2.49) and on average in their fifth semester (*M*=4.54, *SD*=2.02). The latter statistic and prior completion of a clinical traineeship were unevenly distributed between conditions. Moreover, the Lang-Stereotest revealed that all but two participants perceived depth stereoscopically [39], meaning most were able to derive depth information by merging the two perspectives of their eyes. A detailed overview of the sample is provided in table 1 (Tab. 1) and table 2 (Tab. 2).

#### 2.2. Experimental design

**Figure 3:**
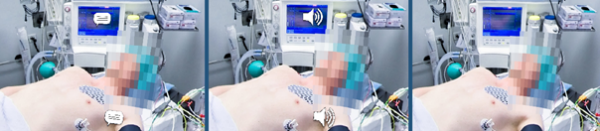
Cropped screenshots of the three virtual tours and their difference in annotation. The cropped screenshots depict the same scene but differ in annotation. From left to right: Hotspots for text boxes, hotspots for audio commentary and no hotspots (handout condition). Hotspots were activated and deactivated with gaze control, i.e. by looking at them for 1.5 seconds.

The virtual tour was created with 360° VR photos (3840x3840 pixels per photo, captured with the 360° camera model Insta360 Pro 2) and designed in three different variations. In a manner of quasi-randomization, study participants were allocated to these variations depending on their date of registration in a predefined sequence. In the first two variants, hotspots were used to link the 360° VR photos and to present information about the facilities, medical devices and clinical procedures either as text boxes (see figure 2 (Fig. 2)) or as audio commentary. In terms of Anderson and Krathwohl's revision of Bloom's taxonomy [16], [17], the given information was factual (discrete elements of information, e.g. a definition of “nosocomial”), conceptual (interrelationship between elements of information, e.g. functional organization of a surgical unit), and procedural (series of steps, e.g. sequence of a surgical hand disinfection) in nature. The hotspots, which were realised as icons of speech bubbles and megaphones (see figure 3 (Fig. 3)), could only be activated once. The length of the individual annotations was 67.36 words on average (*SD*=6.67), which were recorded intelligibly with a moderate average speed of 2.46 words per seconds (*SD*=.21). The speed and volume of the audio commentary as well as the size of the text, which was deemed clearly legible by the authors, could not be modified by the participants. In the third control variant, the same information was not annotated but given separately on a printed handout (H) (see attachment 1 ), which was to be read once before the virtual tour within a time limit of 10 minutes. The participants were instructed to refrain from rereading if they finished early.

**Figure 4:**
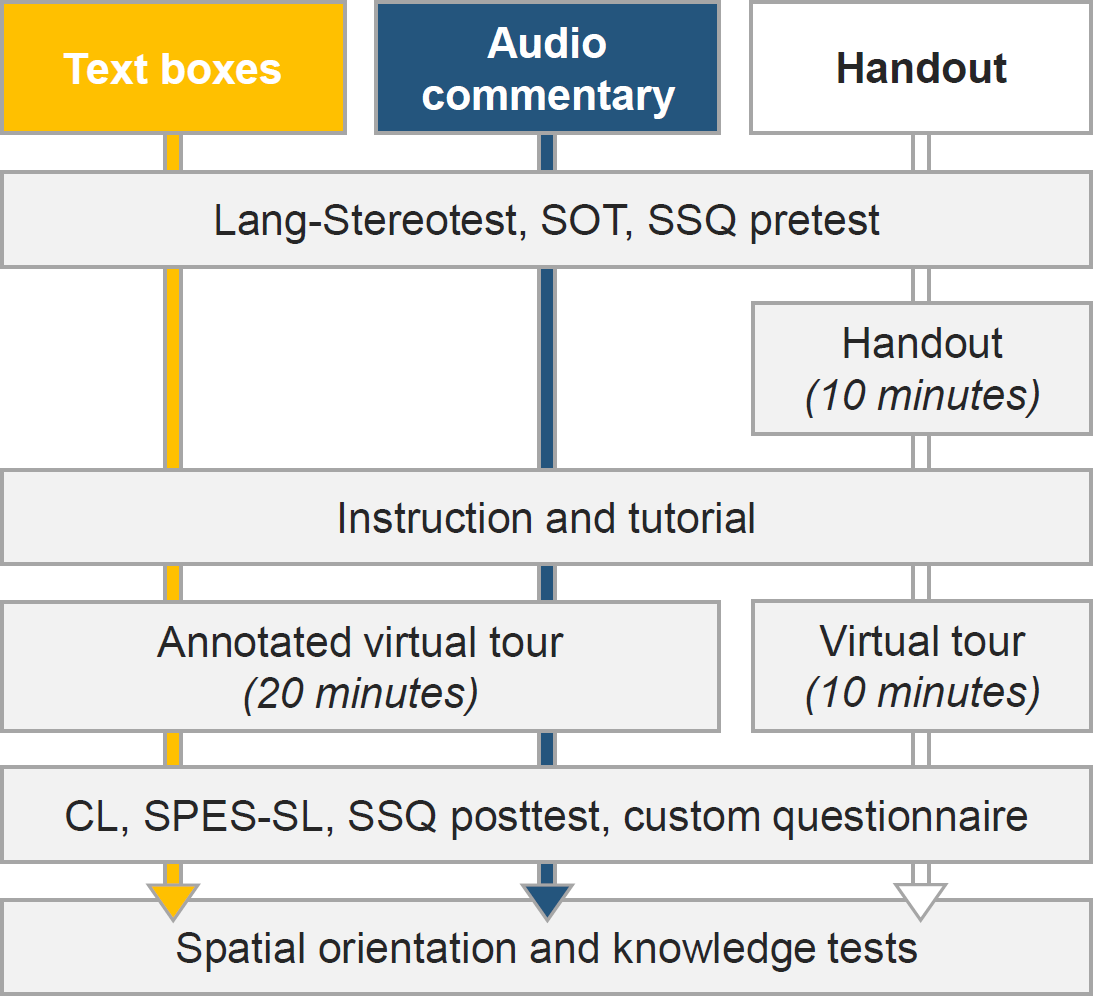
Study design and applied instruments. SOT=Spatial Orientation Test [40], SPES-SL=Spatial Presence Experience Scale – self location [41], SSQ=Simulation Sickness Questionnaire [42], CL=Cognitive load instrument by Klepsch et al. [43].

The students experienced the tour with an HTC Vive HMD (1080x1200 pixels per eye, 90 frames per second, 110° field of view) (see figure 2 (Fig. 2)), which comprises two distinct displays, i.e. one per eye. Stereoscopic vision was achieved by displaying each scene with a slight offset between the two displays. Using gaze control, students activated hotspots and name tags by looking at them for 1.5 seconds. Controller input was therefore absent. To familiarize the participants with the technology, they completed a short tutorial before starting the virtual tour through the surgical unit with a time limit of 10 (handout) or 20 minutes (annotated), respectively. Time limits differed between conditions because annotation prolonged the virtual tour compared to the control group. If a student passed the time limit, she or he was urged to finish rapidly once. Before and after the virtual tour, data was gathered with tests and questionnaires (see figure 4 (Fig. 4)).

#### 2.3. Instruments

##### 2.3.1. Knowledge acquisition

To evaluate knowledge acquisition, a knowledge test was conducted at the end of the study (see attachment 2 ). To avoid recognition instead of recollection, the test consisted of 23 open questions without options to choose. According to Anderson and Krathwohl’s levels of cognitive processing [16], [17] the questions required the participants to remember the presented information (retention; 15 questions) or to demonstrate their understanding of it (comprehension; 8 questions) within a time limit of 15 minutes. The knowledge test did not differentiate between factual, conceptual and procedural information, because the categories could not be distinguished accurately enough. With group affiliation masked, the participants’ answers were assessed by two medical experts, who were guided by a standardized scoring scheme (see attachment 2 ). One of the experts co-authored this article. After scoring the answers independently, the experts discussed discrepancies and reached consensus based on the scoring scheme. The scores were eventually aggregated to a total value, which could be distinguished between retention and comprehension with maximum scores of 41.5 and 23 points, respectively.

##### 2.3.2. Spatial orientation

**Figure 5:**
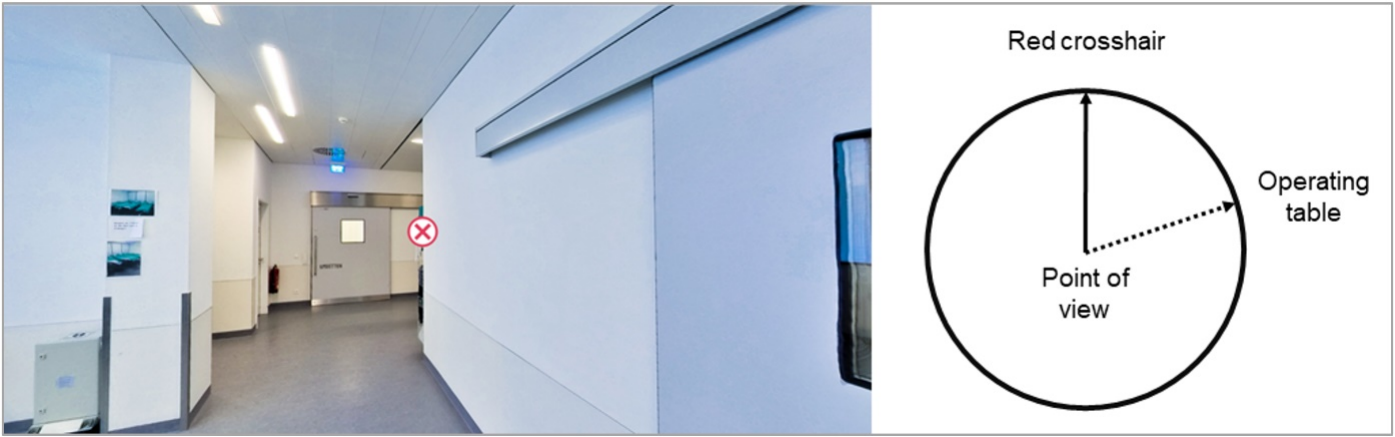
Test for self-location performance as an aspect of spatial orientation. The test was modelled after the Spatial Orientation Test (SOT) by Hegarty et al. [40].

With regard to spatial orientation, two factors were measured. Performance in self-location was measured with a custom adaption of the Spatial Orientation Test (SOT) by Hegarty et al. [40]. After the intervention, students were given eight rectangular screenshots from the virtual tour which were annotated with red crosshairs marking their line of sight. With this reference point being depicted as a line on a piece of paper, the participants were asked to draw another line in the assumed direction of the recently seen operating table (see figure 5 (Fig. 5)). The angular differences between the answers and the correct directions were aggregated and averaged to a self-location performance score ranging from 0 to 180, with a lower score being more favorable.

The second aspect of spatial orientation measured was object-location. Students were given ten extracts of the building plan, depicting different parts of the surgical unit. The students were tasked with locating one object per map among seven positional options each. Correct answers were aggregated to an object-location rating with a maximum score of 10.

##### 2.3.3. User experience

For measuring presence, an authorized German translation of the Spatial Presence Experience Scale (SPES) was administered [41]. The students rated three items concerning their feeling of physical presence on a Likert-type scale ranging from 1 (*“I totally disagree”*) to 5 (*“I totally agree”*). One SPES item referring to observable action was omitted because 360° VR photos are static.

Simulation sickness was measured with the SSQ (Simulation Sickness Questionnaire) before and after the virtual tour [42]. The total SSQ score, which was defined as the increment between the two measurements, expresses the severity of the simulation sickness caused by the virtual tour. According to the authors, a total score of 10 or higher relates to a significant level of discomfort.

The individual cognitive load was measured directly after the virtual tour. According to the cognitive load theory [36], the effort of keeping information in the working memory can be categorized in three different loads. While “intrinsic load” is determined by the complexity and novelty of the input, “extraneous load” is imposed by an adverse mode of presentation. Ideally, extraneous load is minimized to facilitate the cognitive learning process, which in turn constitutes “germane load”. All three types of cognitive loads were measured with the German version of Klepsch et al.’s cognitive load instrument, comprising sevens items in total. Participants rated the items on a Likert-type scale ranging from 1 (*“I totally disagree”*) to 7 (*“I totally agree”*) [43].

Moreover, participants were asked to appraise the didactic value of 360° VR photos and give feedback on their virtual tour (enjoyment and preferred mode of annotation). These items were rated either binary (*“yes”* or *“no”*) or on a Likert-type scale ranging from 1 (*“I totally disagree”*) to 5 (*“I totally agree”*).

##### 2.3.4. Learner characteristics

The participants’ spatial orientation ability was assessed with the unmodified SOT [40], which quantifies the self-location aspect of spatial orientation only. Analogous to its modified version (see chapter 2.3.2), the unmodified SOT yields an average deviation in degrees between 0 and 180. A lower score indicates to a higher spatial orientation ability. In addition, custom items were included to survey sociodemographic and personality variables such as affinity for information technology (IT) and interest in VR and 360° media with a Likert-type scale ranging from 1 (*“I totally disagree”*) to 5 (*“I totally agree”*).

#### 2.4. Statistical analysis

Based on a visual analysis of QQ-plots, normal distribution and homogeneity of variances was assumed for the collected data. Differences in mean values were therefore investigated with *t*-tests for independent samples and one-way ANOVAs. The latter were differentiated with subsequent Fisher’s LSD post-hoc tests. Analyses were two-tailed and p-values below 0.05 were considered to be significant. For effect sizes, partial eta squared (*η*^2^) for ANOVAs and Cohen's *d* for *t*-tests were calculated. Moreover, binominal tests were used to examine relations between categorical variables, whereas reciprocal contingencies between metric variables were tested with Pearson correlations.

## 3. Results

### 3.1. Time

For all participants in the control group, 10 minutes was sufficient for reading the entire handout. Regarding the virtual tour, the majority in the handout (*M*=8.32, *SD*=2.09) and the annotated text box condition (*M*=18.90, *SD*=4.26) reached the end before their respective time limit of 10 and 20 minutes. With annotated audio commentary, however, most participants exceeded their time limit of 20 minutes (*M*=20.76, *SD*=2.50) by 2.20 minutes (*SD*=1.80) on average.

#### 3.2. Knowledge acquisition

**Figure 6:**
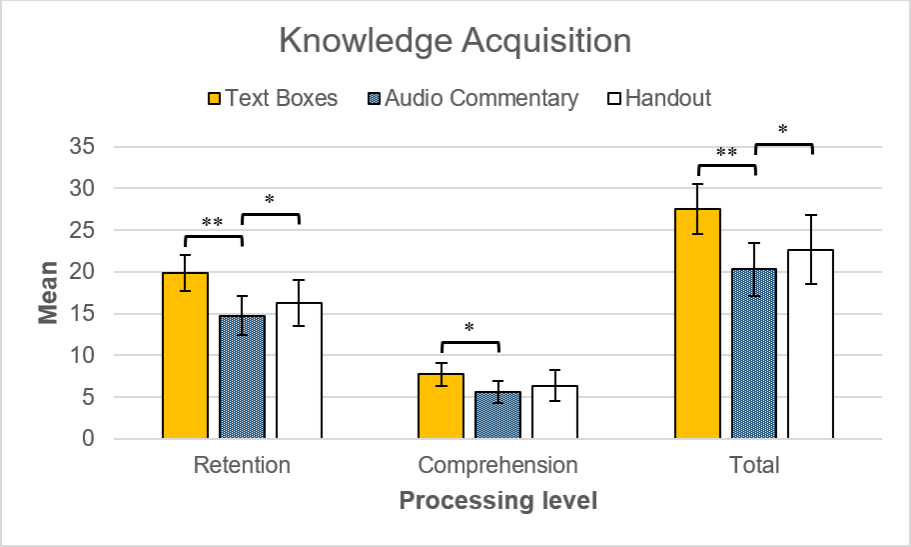
Group comparison of mean values in knowledge acquisition. 95%-CI error bars. Asterisks denote *p*-values of LSD post-hoc tests. The maximum scores for retention (41.5) and comprehension of knowledge (23) amount to a total maximum score of 64.5 points. *p<.05. **p<.01. ***p<.001

The mean overall test score was significantly higher in the annotated text box condition (*F*(2,57)=5.13, *p*=.009, *η^2^*=.15) than in the annotated audio commentary (*p*_LSD_=.003) and the handout condition (*p*_LSD_=.042) (see figure 6 (Fig. 6)). The group who read text in the virtual tour performed best on both levels of cognitive processing even though the margin between annotated and printed text was insignificant for questions requiring understanding (*p*_LSD_=.195). Despite performing worse than the annotated text box condition, the group who listened to the annotated audio commentary deemed 360° VR photos more suitable for knowledge acquisition (*t*(39)=2.48, *p*=.017, *d*=.79). Moreover, 83% of the participants who learned with some form of annotation believed or rather believed that recall in the real surgical unit is improved if information is integrated in the virtual tour.

#### 3.3. Spatial orientation

**Figure 7:**
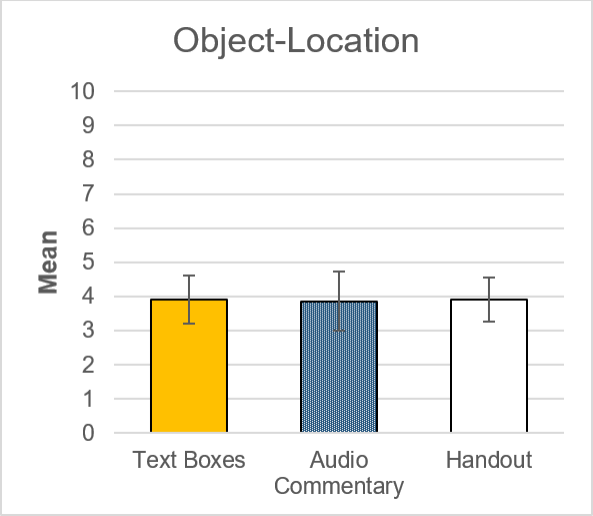
Group comparison of mean values in object-location. Object location ranges between 0 and 10, with the latter representing a perfect score. 95%-CI error bars.

**Figure 8:**
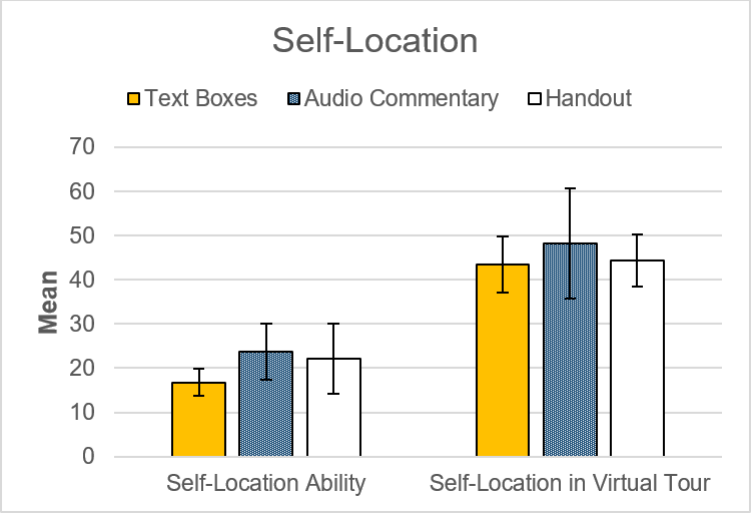
Group comparison of mean values in self-location. Self-location ranges between 0 and 180 degrees of average deviation, with a lower degree being more favorable. Significant differences between groups were absent. 95%-CI error bars.

Concerning performance in object-location (*F*(2,58)=.00, *p*=.996, *η^2^*=.00) (see figure 7 (Fig. 7)) and performance in self-location (*F*(2,58)=.37, *p*=.693, *η^2^*=.01) (see figure 8 (Fig. 8)) no significant group differences were found.

#### 3.4. User experience

For simulation sickness (*F*(2,58)=.38, *p*=.69, *η^2^*=.01) and presence (*F*(2,58)=1.66, *p*=.199, *η^2^*=.05), no significant group differences were found either. Both factors also did not correlate significantly with the knowledge and spatial orientation test scores, even though 28% of the participants across conditions experienced significant levels of discomfort. On a descriptive level, however, annotated audio commentary (*M*=4.32, *SD*=.70) allowed for a higher presence score than the annotated (*M*=3.85, *SD*=.83) and printed text conditions (*M*=4.1, *SD*=.92), and was deemed less disruptive than text boxes (*t*(39)=1.80, *p*=.080, *d*=.56).

**Figure 9:**
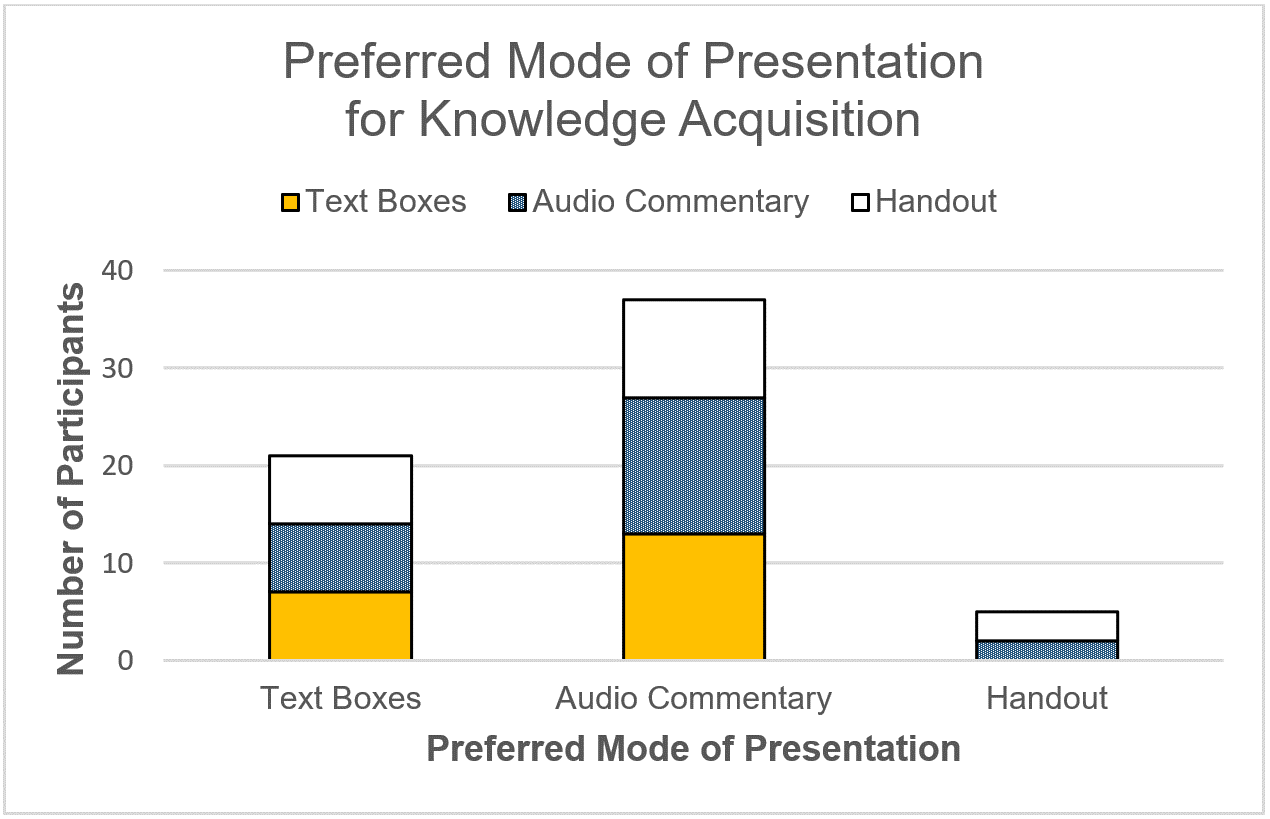
Group comparison of preferred mode of presentation.

While no significant differences in cognitive load were found between the two modes of annotation, annotated audio commentary was evaluated more favorably in general (see table 3 (Tab. 3)). Audio commentary scored descriptively higher on enjoyment and presumed didactic value across conditions. Moreover, reading text in the virtual tour proved to be more difficult than understanding the annotated audio commentary acoustically (*t*(39)=5.56, *p*<.001, *d*=1.74). The control group, which read the information before the virtual tour on a printed handout (H), reported a descriptively higher extraneous load and a significantly lower germane load (*F*(2,58)=4.16, *p*=.02, *η^2^*=.13) than the annotated audio commentary (AC) (*p*_LSD_=.014) and annotated text box condition (TB) (*p*_LSD_=.017). In line with these results, binominal tests showed that most participants preferred to acquire knowledge within the virtual tour (*p_TB_*<.001; *p_AC_*<.001; *p_H_*=.003), with annotated audio commentary being their favoured mode of annotation (see figure 9 (Fig. 9)). 

#### 3.5. Learner characteristics

Self-location ability (see figure 8 (Fig. 8)), affinity for IT, interest in VR and 360° media, and prior usage of VR (see table 2 (Tab. 2)) were not associated with performance in the knowledge or spatial orientation tests. However, interest in VR and 360° media correlated positively with enjoyment (*r*=.37, *p*<.001), presence (*r*=.46, *p*<.001) and presumed didactic value of 360° VR photos (*r*=.46, *p*<.001). 

## 4. Discussion

With 360° VR photos, medical educators can grant immersive insights into regional practices for early clinical exposure. To determine how 360° VR photos should be applied and designed to facilitate learning, three hypotheses were tested in this study:

### Hypothesis 1: Providing 360° VR photos as visual reference improves retention and comprehension of information

Providing 360° VR photos as visual reference does improve knowledge acquisition as previous research suggested [21], [22], but the choice of modality is significant (see 3^rd^ hypothesis). The students’ subjective appraisal confirms the effectiveness of 360° VR photos, with the majority believing that they facilitate subsequent recall in real life. Similarly to previous research on 360° VR videos [11], [18], [19], enjoyment and presumed didactic value of 360° VR photos was high in all variants of the virtual tour. Therefore, their application for early clinical exposure would likely be welcomed by students and convey information effectively depending on their design.

#### Hypothesis 2: The annotation of text boxes in 360° VR photos compromises spatial orientation and presence

Concerning spatial orientation and presence, annotated text boxes did not impair the learners to a significant extent. This is unexpected because the text boxes were deemed more disruptive than the audio commentary. For presence, one explanation might be a missing benchmark as 72% of the participants had no previous VR experience. Having never experienced higher levels of immersion, they may have overrated their sense of being in the displayed surgical unit [44]. Thus, annotated text boxes may still have a diminishing effect on presence but at least less experienced VR users do not mind. With respect to spatial orientation, reading appears to be no hindrance as long as learners have enough time to explore the 360° VR photos in between texts.

#### Hypothesis 3: Annotated audio commentary is superior to annotated text boxes in terms of cognitive load and knowledge acquisition

According to the modality principle [35], text boxes should lead to a higher extraneous load and a lower learning effectiveness compared to audio commentary. However, the knowledge test results are inverse to this prediction even though participants in the annotated text box condition were at a potential disadvantage due to their lack of previous clinical traineeships and to being in a lower semester on average. Annotated text boxes outperformed annotated auditory annotation and the handout condition in both retention and comprehension. Despite spending the most time in the virtual tour, the annotated auditory annotation exhibited the lowest test scores on average. This unexpected outcome can be explained by the learning content. First, the visual reference may not have been essential enough for understanding the presented information. The modality principle applies when two sources of information need to be simultaneously processed and mentally integrated [35]. If students could ignore the displayed environment in large part and focus on reading, the annotated text boxes presumably did not cause cognitive overload. Second, the annotated text boxes could be reread as long as the hotspot was activated, whereas the annotated audio commentary was transient. According to Leahy and Sweller [45], this advantage is particularly impactful with lengthy material. Students may have struggled to adequately process the 67 words on average before the audio commentary ended. Therefore, the finding that audio commentary is disadvantageous for knowledge acquisition might only apply to long texts which cannot be accessed in written form.

The given explanations are not supported by the conducted cognitive load measurements. The inconsistency is likely caused by the subjective nature of the self-report instrument. Learners may not be able to discern the three types of cognitive load and have different internal standards for evaluating cognitive effort [46], [47]. This assumption is substantiated by the significantly higher extraneous load in the handout condition. Without any annotated information, students could focus exclusively on spatial orientation. In theory, this should have resulted in a lower extraneous load. Regarding the other factors of user experience, annotated audio commentary was the favoured option on a descriptive level. Students reported greater feelings of presence and enjoyment with auditory annotation. Even though it prolonged the virtual tour, most would choose annotated audio commentary over annotated text boxes and a handout when given the chance. Thus, annotated audio commentary is recommended, if knowledge acquisition is not a priority or the information is accessible in written form, too.

Simulation sickness has afflicted almost a third of the participating students across conditions but did not impair neither learning nor spatial orientation performance. The relatively high prevalence might be explained by the fact that the students did not see themselves in the 360° VR photos and appeared to float in mid-air. If the learners were represented as virtual avatars, simulation sickness might decrease. With respect to the measured learner characteristics, no determinant was found for the effectiveness of 360° VR photos for knowledge acquisition and spatial orientation. However, students with an interest in VR and 360° media tend to immerse and enjoy themselves more in 360° VR photos and ascribe a greater didactic value to them. These findings conform with previous research and imply that a greater application of 360° VR photos in medical education would be followed by a higher demand and appreciation of the medium [30], [31], [32], [33]. 

Naturally, the findings of this study must be interpreted in regard of its limitations. Apart from [21], the combination of 360° photos and HMDs has been given little attention in research so far. To validate the reported findings, they must be replicated with discretely measurable knowledge categories and greater sample sizes exhibiting a higher variance in familiarity with VR. Moreover, the knowledge and spatial orientation tests were conducted shortly after the virtual tour. The results therefore only demonstrate the short-term effectiveness of 360° VR photos. Lastly, measuring cognitive load physiologically with an EEG and eye-tracking in the HMDs would have helped to explain the findings more conclusively [48], [49]. Future studies should use these methods to further explore how cognitive capacity may be directed and efficiently used in 360° VR photos. In doing so, the quality of the annotated information should be considered. Apart from its length, the learning experience might also be influenced by the speed or volume of the audio commentary. In addition, the effectiveness of 360° VR photos should be compared with learning scenarios in real life such as an in-person tour through the surgical unit.

## 5. Conclusion

360° VR photos are a low-threshold and cost-efficient possibility to facilitate clinical exposure in the early stages of medical training. Apart from granting immersive insights into the daily clinical routine, the medium can convey information and a sense of spatial orientation effectively in the same learning scenario. While its educational application is generally well-received by students, those with an interest in VR and 360° media immerse and enjoy themselves more in 360° VR photos than others and ascribe a higher didactic value to them. Contrary to the authors’ expectation, the ideal mode of annotation is not dictated by Mayer’s modality principle. For information like in this study, annotated text boxes are better for knowledge acquisition than the subjectively preferred audio commentary. This finding, however, is probably contingent on the length and the quality of the annotated text. To identify boundary conditions and validate the findings, more research is required on the design and educational use of 360° VR photos.

## Notes

### Funding details

This work was supported by the committee of educational research at the Medical Faculty of Ulm University.

#### Ethics committee

The ethics committee of Ulm University determined that the study required neither legal nor ethical approval.

## Competing interests

The authors declare that they have no competing interests. 

## Supplementary Material

Handout

Knowledge test and scoring scheme

## Figures and Tables

**Table 1:**
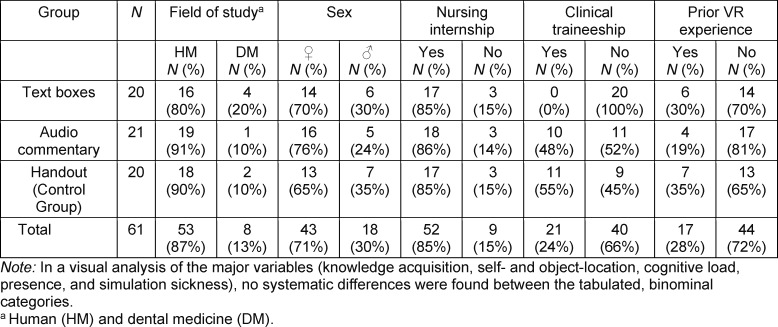
Nominal sample description

**Table 2:**
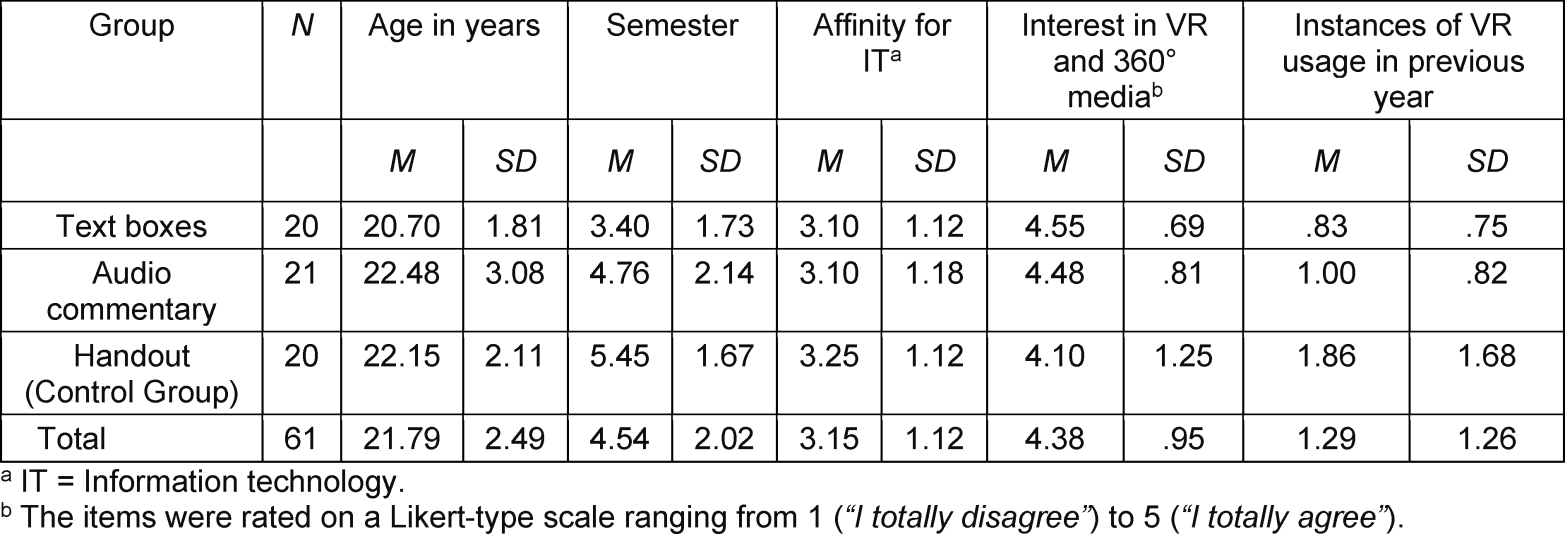
Metric sample description

**Table 3:**
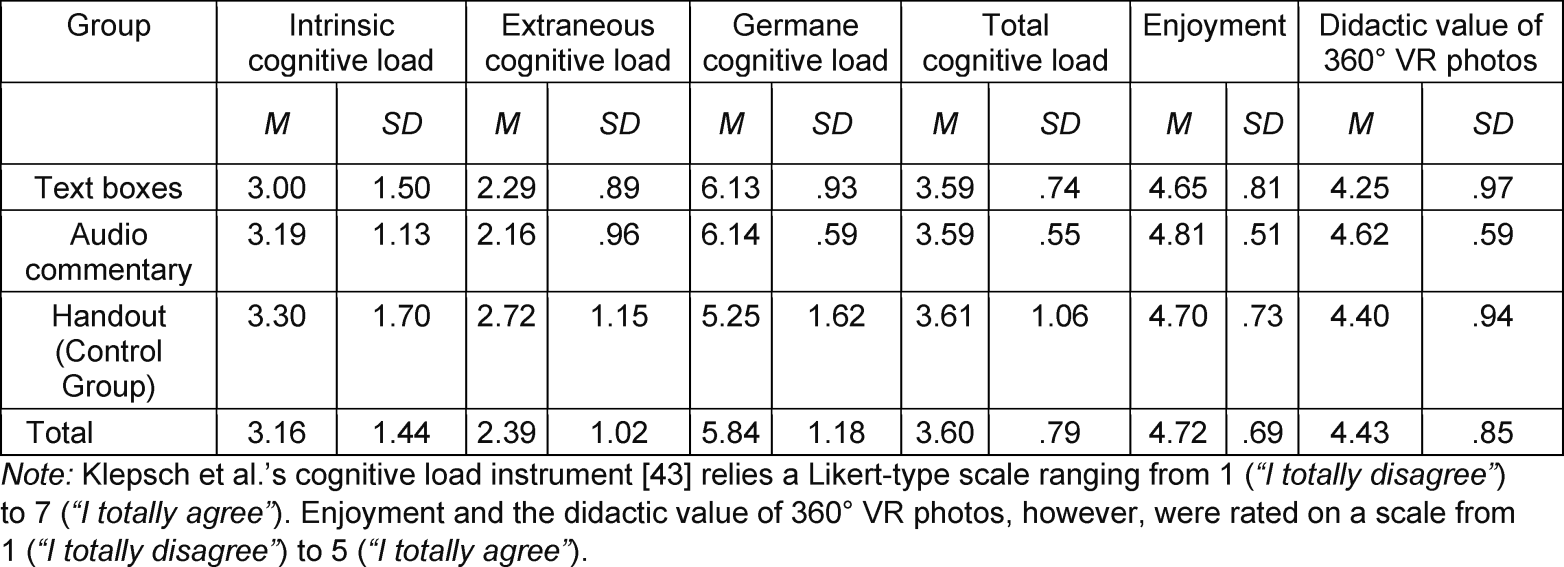
Descriptive statistics of cognitive load measurements and subjective appraisals
