# Supplementary material for: Immersive medium for early clinical exposure – knowledge acquisition, spatial orientation and the unexpected role of annotation in 360° VR photos
Source: GMS J Med Educ. 2023 Feb 15;40(1):Doc8. doi: 10.3205/zma001590 (PMC10010766; doi:10.3205/zma001590)
Supplement: Handout [file JME-40-8-s-001.pdf]

Attachment 1: Handout

The operating area

This handout describes the structure of the operating area of the University Hospital Ulm and the way in which it functions. The order of the sections is based on the order of the rooms, which you will visit afterwards as part of a virtual tour.

1. Changing room (unclean)

In the unclean changing area of the personnel transfer area, staff must remove any street or hospital clothing that may be contaminated with microorganisms from the hospital or the environment down to the undergarments and store it. Visible jewelry on hands, wrists, and around the neck must also be removed for hygiene reasons. The only exception are stud earrings which are covered by the surgical cap.

Hygienic hand disinfection must be performed before entering the clean operating area in order to protect patients from nosocomial germs and infections, i.e., those acquired in the hospital. The following procedure must be followed:

1. Wash hands and forearms with warm water.
2. Spread approximately 5 ml of soap solution onto hands and forearms and lather up.
3. Rinse hands thoroughly, draining water from distal to proximal areas.
4. Dry hands and forearms thoroughly with disposable towels.
5. Wet dry hands with disinfectant for 30 seconds.

2. Changing room (clean)

Clean surgical clothing – consisting of pants, shirts, and shoes – is sorted by size on laundry racks in the clean changing room. This clothing is green in color and may only be worn in the OR. Exceptions include patient transports to the intensive care unit and stays in the trauma room, when a blue gown is worn on top.

Blue clothing is intended exclusively for use in outside areas or in "septic ORs." Patients with infected wounds are cared for in septic ORs. Since these wound pathogens must not be spread further, the different clothing color serves as a visual warning signal.

3. Dictation room

The surgery report, in which every step of the surgery is recorded, is dictated in the dictation room. Details documented include, for example, the type of incision, the condition of the surgical site, the surgical measures performed, and the type of wound closure applied. The surgery report also contains information on the surgical team performing the procedure, the chronological sequence of the surgery, and any particularities that must be observed postoperatively. Information on implants used, such as the type and serial number of the implant and the date of implantation, is also recorded in the patient's implant passport.

One the one hand, detailed surgical documentation is mandatory for legal reasons; on the other hand, it serves to provide information about the surgical findings and the surgical steps performed at any time, even in retrospect.

4. Transfer

During transfer, patients are moved from the bed to the operating table and transferred to the operating area. The following details are checked

as soon as the patient arrives, even before the patient transfer begins:

- Patient identity
- Type and site of the procedure
- Presence of written surgical and anesthesia consent
- Marking of the surgical site
- NPO status
- Allergies
- Completeness of the patient file

There are two options for the transfer itself: The patient can either slide over from their bed to the operating table on their own or be transferred with the help of the stationary patient transfer unit. If the patient changes beds on their own, they should not have to get up to do so, as premedications can cause orthostatic problems which may result in issues such as circulatory collapse. The use of the stationary patient transfer unit, also known as the Transmaquet, is recommended especially for patients with existing unstable and/or painful conditions. It is computer-controlled and its skin-friendly, disinfectant-resistant conveyor belt is preheated to approximately 30° Celsius.

The stationary patient transfer unit is operated using an infrared remote control. The colors on the buttons of this remote control mark the directions in which the unit moves: orange stands for transfer out and green for transfer in. If the remote control fails, it is also possible to operate the unit using the accompanying control panel.

5. Scrub room

Prior to surgery, the entire surgical team must perform surgical hand disinfection. The following instructions must be observed:

- Fingernails must be unpainted and cut short.
- There must be no nail bed injuries or inflammation.
- Scrubbing of hands and forearms should be avoided due to skin irritation and increased risk of pathogen transmission.
- Faucets and dispensers should not be operated with the hands, but with the elbow.

For surgical hand disinfection, dry hands and forearms are rubbed with an alcohol-based disinfectant from distal to proximal areas and kept moist for the entire exposure time of 3 to 5 minutes. The following sequence should be followed during disinfection:

1. Wet the hands.
2. Wet the forearms up to and including the elbows.
3. Disinfect the hands repeatedly with arms, hands, and fingertips pointing upwards.
4. Hold the hands in an upright position at chest level.

6. Induction

The selected anesthetic procedure is prepared and performed in the induction room. This procedure may be regional, spinal or general anesthesia. During general anesthesia, consciousness, pain sensation, memory, and often muscle tension are inhibited. Inhaled and intravenous anesthetics, as well as opioids and muscle relaxants, are used for this purpose.

An important prerequisite for the safe application of general anesthesia is ensuring respiratory function. This is done either by mask ventilation, a laryngeal mask or endotracheal intubation. A distinction is made between controlled and assisted ventilation. While spontaneous breathing is completely suspended during controlled ventilation, the assisted method involves adapted ventilation, i.e., the patient's own breathing is supported using equipment or manually.

Attachment 1: Handout

One feared complication during anesthesia induction is aspiration, the risk of which is particularly high in pregnant, non-NPO or very obese patients. Aspiration causes patients to vomit after the respiratory reflexes have already been inhibited. The vomit can therefore enter the trachea and bronchi and induce severe tissue damage. This damage then triggers aspiration syndrome or Mendelson syndrome, which leads to respiratory failure and is associated with a high lethality. Lethality refers to the ratio of deaths to the number of acutely affected individuals.

Defibrillators are installed in induction rooms where patients with circulatory instability are frequently treated. If life-threatening ventricular fibrillation with circulatory arrest occurs in a patient, immediate defibrillation is indicated as a lifesaving measure. Defibrillation refers to a shock-like delivery of current to the heart, which interrupts fibrillation and restores the majority of the heart muscle cells to the same excitation phase. This prompts the sinus node, the heart's natural pacemaker, to resume regular stimulation.

If circulatory arrest is to be expected, special adhesive electrodes are attached to the patient in such a way that the current can flow effectively through the heart axis, e.g., bilaterally on both sides of the thorax or also only on the left side of the thorax in a sternal-apical or ventral-dorsal direction.If the patient has a pacemaker, the electrodes must be positioned at least 8 cm away from the unit and the current flow should be as perpendicular as possible to the presumed pathway of the pacemaker cable.

The recommended amount of energy delivered during defibrillation is as follows:

- 360 joules for monophasic shocks.
- 150 to 200 joules for the first shock, 150 to 360 joules for all subsequent shocks in biphasic shock deliveries.
- For children, the amount of energy for both monophasic and biphasic defibrillation is determined by body weight. The recommended amount is 4 joules per kilogram.

7. Operating room (OR)

After entering the operating room, sterile surgical gowns and surgical gloves are put on the surgical team by the "circulator". The gloves are put on in such a way that they extend over the lower edge of the sleeves of the surgical gown. Special precautions, such as wearing protective goggles, must be taken for patients and surgeries with an increased risk of infection or injury.

To prepare the patient, the incision area is cleaned and disinfected using skin antiseptics. Alcohol-based preparations are always the first choice because of their high and rapid efficacy. The addition of antiseptics with a residual effect, such as chlorhexidine or octenidine, can offer an additional benefit in surgeries lasting more than two hours. For gynecological surgeries and surgeries involving the mucosa, mucosa-friendly antiseptics must be used instead of alcohol-based ones.

Perioperative antimicrobial prophylaxis must be given between 60 minutes before and just prior to skin incision. Repeated administration of the antimicrobial should be performed only for very long surgeries or when large quantities of blood are transfused. Antimicrobial prophylaxis usually involves administering second-generation cephalosporins. In abdominal surgery, an antibiotic that is effective against anaerobes is also used. The continuation of perioperative prophylaxis for days has no additional protective effect.

Transection of tissue as well as coagulation of smaller blood vessels is performed using electrocautery. To allow the electrocautery

current to flow, the patient is grounded with a neutral electrode close to the surgical site. If the contact area of the neutral electrode is insufficient, the electrocautery pen will sound an alarm. When using electrocautery, care must be taken to ensure that the patient is not wet, otherwise the flow of current will cause serious burns.

Before the surgery begins, a final check is performed in the form of a team time-out. The following points are checked against a list:

- Is this the right patient?
- Which surgery is to be performed and which side is the surgery to be performed on?
- Are all preliminary findings that are important for the surgery known?
- Is banked blood available if needed?
- Does the patient have any allergies or other special risks such as cardiovascular diseases?
- Does the patient require antibiotics?
- Are all the materials needed for the surgery present?

Heat management in the OR is very important since the patient's ability to regulate body temperature is reduced significantly during anesthesia and the room temperature in the OR is low. Heat-maintaining procedures include, for example, warmed terry cloth sheets as well as warming mats and warming blankets with warm air flowing through them. It should be noted that infants and young children lose body heat much more quickly than adults due to their metabolic situation and their disproportionately large body surface area. Temperature can be monitored via the ear canal, the esophagus or temperature probes. The latter are often connected to a urinary catheter, but are sometimes also inserted rectally.
