# Supplementary material for: Immersive medium for early clinical exposure – knowledge acquisition, spatial orientation and the unexpected role of annotation in 360° VR photos
Source: GMS J Med Educ. 2023 Feb 15;40(1):Doc8. doi: 10.3205/zma001590 (PMC10010766; doi:10.3205/zma001590)
Supplement: Knowledge test and scoring scheme [file JME-40-8-s-002.pdf]

Attachment 2: Knowledge test and scoring scheme

Classification of questions

Bloom level: remembering 1
1. Open questions (0.5 points per partial answer) 1
2. Closed questions (1 point per partial answer) 2
3. Definitions (2 points per answer) 3

Bloom level: understanding 3
4. Explanation (2 points per answer) 3
5. Transfer questions with list (1 point per partial answer) 4
6. Case-based transfer questions (1 point per partial answer) 4

Answers that are partially correct will be awarded 50% of the points available. This does not include answers that are worth 0.5 points.

Bloom level: remember

1. Open questions (0.5 points per partial answer)

a_bericht *What information is documented in the surgery report?*
List as many examples as possible.

- Incision type
- Condition of the surgical site
- Surgical measures
- Wound closure type
- Information about the surgical team
- Chronological sequence of the surgery
- Particularities to be taken into account postoperatively
- Implant type
- Implant serial number
- Date of implantation

Attachment 2 to Speidel R, Schneider A, Walter S, Grab-Kroll C, Oechsner W. *Immersive medium for early clinical exposure – knowledge acquisition, spatial orientation and the unexpected role of annotation in 360° VR photos*. GMS J Med Educ. 2023;40(1):Doc8. DOI: 10.3205/zma001590

a_check *Which aspects are checked in the operating room before each procedure? List as many examples as possible.*

- Is this the right patient?
- Which surgery is to be performed?
- Which side is the surgery to be performed on?
- Are all preliminary findings that are important for the surgery known?
- Is banked blood available if needed?
- Does the patient have any allergies?
- Does the patient have any other special risks such as cardiovascular disease?
- Does the patient require antibiotics?
- Are all the materials needed for the surgery present?

2. Closed questions (1 point per partial answer)

a_zeit *What is the minimum amount of time for which hands should be kept moist with disinfectant during hygienic hand disinfection?? How long during surgical hand disinfection?*

- Hygienic: 30 seconds
- Surgical: 3-5 minutes *(3, 4 or 5 is correct)*

a_kleidung *Where, or under what circumstances, may green surgical clothing be worn?*

- In aseptic ORs
- During patient transports to the intensive care unit
- When working in the trauma room with blue gown

a_anaesthesie *Which anesthetic procedures do not fall under the term "general anesthesia"?*

- Regional anesthesia
- Spinal anesthesia

a*_vollnarkose* *Which mental functions are always inhibited during general anesthesia?*

- Consciousness
- Pain sensation
- Memory

a_atemfunktion *What can be used to ensure respiratory function during general anesthesia?*

- Mask ventilation/ laryngeal mask
- Endotracheal intubation

a_herzschrittmacher *What must be observed when attaching adhesive defibrillation electrodes if the patient is dependent on a pacemaker?*

- 8cm distance from the unit
- Current flow should be as perpendicular as possible to the presumed pathway of the pacemaker cable

a_verhaeltnis *What is the ratio between the amount of energy and body weight when defibrillating children?*

- 4 joules per kilogram

a_temperatur *Where can the patient's temperature be measured during surgery?*

- Auditory canal
- Esophagus
- Urinary catheter
- Rectally

a*_risiko* *Name the risk factors for aspiration.*

- Pregnancy
- Non-NPO status
- Obesity

a_umbettanlage What are the characteristics of the stationary patient transfer unit?

- Computer-controlled
- Skin-friendly
- Resistant to disinfectants
- Preheated

3. <u>Definitions (2 points per answer)</u>

a_nosokomial *What does the term "nosocomial" refer to?*

- Germs and infections acquired in the hospital.

a_letalitaet *Define the term lethality in a few words.*

- The ratio of deaths to the number of acutely affected persons.

a_schutzbereich *What characterizes the "sterile area" in the operating room?*

- Microbial contamination is reduced in the protected area by means of ventilation technology.

Bloom level: understand

4. <u>Explanation (2 points per answer)</u>

v_schockraum *Why must a blue gown be worn over green scrubs while working in the trauma room?*

- The blue gown serves as a visual warning signal that newly arrived patients are potentially septic.

v_kinder *Why do children and infants lose body heat significantly faster under general anesthesia than adults?*

- Metabolic situation
- Disproportionately large body surface

5. Transfer questions with list (1 point per partial answer)

v_konsequenzen *List the negative consequences that can potentially result from an incomplete surgery report.*

- Surgeon is unable to defend themselves in the event of a lawsuit
- Surgeon faces legal consequences as they are obligated to maintain documentation.
- Patient cannot be fully informed about the procedure.
- Subsequent physicians are unable to access information.

v_voraussetzungen *List the prerequisites that must be met for properly performed hygienic hand disinfection to be effective.*

- Unpainted fingernails
- Short fingernails
- No infections
- No nail bed injuries
- No jewelry worn on hands

6. Case-based transfer questions (1 point per partial answer)

v_fraktur *How do you transfer a patient with a clavicle fracture in the patient transfer area? Why are the two alternatives not an option?*

- Using the stationary patient transfer unit
- Reasons
  - Premedication could cause orthostatic problems when the patient gets up
  - Sliding over on their own is too painful due to the fracture and could lead to further tissue damage.

v_tumor *Severe bleeding occurs during gastroenterological tumor removal. What does the type of surgery and bleeding indicate with regard to perioperative antimicrobial prophylaxis?*

- Repeated administration of antimicrobial agent
- Administration of an antibiotic that is effective against anaerobes

v_verbrennung *A patient complains of burns to the skin on his back after a surgical procedure. What could have caused these?*

- Patient was wet during use of electrocautery

v_antiseptikum *What should ideally characterize the antiseptic used during a hysterectomy that lasts several hours?*

- Low irritant effect on mucous membranes
- Residual effect
